# Under the Red Cross: A Record of Wonderful Achievements

**Published:** 1915-10-30

**Authors:** 


### UNDER THE RED CROSS.

### A RECORD OF WONDERFUL ACHIEVEMENTS.

To commemorate the anniversary of the union of the British Red Cross Society and the Order of St. John, the *Times* on October 21 issued a remarkably interesting supplement of thirty-two pages dealing exclusively with the work of these two Societies, and their joint achievements during the present campaign. In The Hospital of October 23 (p. 86) we published a brief article devoted to the same subject, but in view of the exhaustive and clear account of the many activities of the Joint Committee which the *Times* has presented to its readers we cannot refrain from returning to the matter. We recommend all who are interested in the great work which the united organisations are doing to obtain, if possible, a copy of this *Times* Supplement. One can imagine no surer means of appealing to the public for support in this wonderful movement than by bringing home to them the facts and realities of the work of the Red Cross.

### The Origin of the Red Cross.

The precise date of the origin of the Red Cross was the war in Italy in 1859. Before that date private charity, it need hardly be said, had been called in on more than one occasion to help the army authorities in various countries in the work of caring for the wounded. But these efforts were spasmodic, and the experience gained was not available for future use. Armies, meantime, had their own medical services, but the revelations made during the Crimean War by Sir William Russell in the columns of the *Times*, and the subsequent mission of Florence Nightingale, dispelled the illusion that the Army Service was of itself sufficient to cope with the various emergencies which were sure to occur. The experiences in the Crimea were destined to be repeated in Lombardy in 1859, and it is recalled by the *Times* that M. Henri Dunant, in his account "Un Souvenir de Solferino," recorded a small portion of these scenes and so forced public attention to see the defects of the Sanitary Service and the need for remedying them. He called for an auxiliary service, and demanded that it should be authoritatively recognised and properly disciplined. Three months after the publication of this book the inferences gathered from it were made the subject of a discussion at a meeting of the Société Genevoise d'Utilité Publique, held on February 9, 1863. A committee was formed which set about the task of collecting and framing a comprehensive scheme, and it was decided to call an international conference, which assembled at Geneva in October of the same year. The findings of this conference formed the basis of all future Red Cross work, and the Convention of Geneva was signed within six months by eight European States, and has since been accepted by very many more. In England the Red Cross did not become active until the Franco-Prussian War, when there arose a great desire in this country to offer some help to the wounded of both belligerents. As a direct result of a letter to the *Times* from Lord Wantage, a national committee was formed, and the Prince of Wales (King Edward VII.) consented to become its President. Space will not permit us to follow any further the *Times* article on the developments of the movement, from its origin to the time when, on the outbreak of the present war, the British Red Cross Society called together its various agencies to help the War Office, and rather more than two months later received the co-operation of the Order of St. John.

### The Red Cross at the War.

Concerning the early work of the Red Cross in France and Belgium the account is indeed thrilling. As we all now know the Society was not found wanting when the call came. The first party left England on August 12, eight days after the outbreak of war, and proceeded at once to Brussels. Before the fall of the city, which occurred so soon, six parties had been sent to Brussels. The account then takes us to those terrible days of the retreat from Mons, and shows how the Red Cross Society helped to save the situation by its splendid work. We read of the truck trains without brakes in which the wounded, lying upon dirty straw, were jolted and jostled through long days and nights; how the congestion of traffic caused the holding up of the railway service often for long periods; and how as a result of advices of these matters reaching headquarters from the Commission that was actually on the spot great new activities began in London which have led to the accomplishment of marvellous things—the establishment of hundreds of auxiliary hospitals, the sending out of doctors and nurses, the supplying of hospital equipment, the provision of motor-ambulances, and so on. The constant changes of the British base during that period made it necessary to change the *locus* of the hospital units, and rendered the opening stages of the Society's work extraordinarily difficult. But the battle of the Marne at the beginning of September enabled the Red Cross to get to work in Paris.

### Boulogne a Hospital City.

Describing the arrangements that had to be made after the battle of the Aisne, when the greatest battle of British history was being fought some sixty miles from Boulogne, while the British bases were situated hundreds of miles away at Havre and St. Nazaire, and when the hospitals at these places and in Paris were full and Boulogne had been evacuated and was empty of all equipment and stores, it is related how the Joint Committee helped the R.A.M.C. to transform the whole situation in Calais and Boulogne. The wounded were coming in at the rate of 3,000 to 4,000 a day both to Calais and Boulogne. Tremendous demands were made upon the Society; it alone was in a position to supply equipment. Thanks to these stores, to the *personnel* which accompanied them, and to the magnificent labours of the R.A.M.C., Boulogne became converted into a great hospital city during the days of the first battle of Ypres. The R.A.M.C. established hospitals in the Casino and in various hotels, and to these the Red Cross gave and lent freely. Ambulance-cars were hurried out to the seat of war as fast as possible, and soon a great convoy of cars was at work taking the wounded from the station to the various hospital ships. The Red Cross Organisation undertook the whole work of clearing the trains during the terrible days of October 1914, when the British Army held and defeated all the German attempts to break through to Calais, and it is recorded that the hardest day's work in that early period was the removal of 3,687 wounded with only twenty-five cars—1,694 from the trains to the hospitals, and the remainder from the hospitals to the ships.

The Role of the Motor Ambulance.

Among the greatest of the achievements of the Joint Committee is the work of ambulance transport. To-day there are some 650 motor ambulances at work in France. The official figures for March, the month of Neuve Chapelle, showed that 33,941 patients were taken from train to hospital and from hospital to ship. Of these 30,428 went by Red Cross car, and each car travelled thirty-one miles a day. "The cars," says the writer, "are the feature of the streets of the little seaport (Boulogne). You see them everywhere all day long, and all night long too, those going to the station rolling swiftly over the pavement, and those returning creeping at a snail's pace for the sake of the living freight, to which every jolt and jar is a stab of pain. The sight is great and inspiring; as the days pass you realise the magnitude of this effort, and, no less clearly, the wonderful smoothness with which the organisation works. These cars are absolutely essential. . . . They have cut the burden of the wounded man in half; they have saved innumerable lives, and have eased untold sufferings."

Red Cross Hospitals in France.

In all, the Red Cross Society has upwards of 1,000 beds in France available for British wounded. In addition some special institutes of great importance and interest are administered. The hospitals are staffed by the Joint Committee working in conjunction with the Army authorities. "They are," says the writer, "all of them places of healing in the truest sense of the word, places where scientific knowledge goes hand in hand with sympathy and kindness, and where a wounded and suffering soldier finds the atmosphere of rest and peace so blissful after the moil and weariness of the battlefield." The location of the eight Red Cross hospitals in France was stated in The Hospital article last week, but far larger than any of them is the St. John's Brigade Hospital of 520 beds at Etaples, which is the special work of the Order of St. John.

Other Achievements.

Of the activities of the Red Cross in Gallipoli, of the work of the Joint Committee in Serbia, Montenegro, Italy, and Russia, of the way in which the British Empire beyond the seas is helping this great cause, of the efforts of the Society to trace missing soldiers; and, in fact, of all the branches of the Society's work, we are given long and interesting narratives in the *Times* Supplement. There are also special articles dealing fully with King George Hospital, Netley Hospital, the St. John's Brigade, the Friends' Ambulance Unit, the Red Cross in England, Voluntary Aid Detachments, Gifts to Prisoners, and the Red Cross Library and Hospitals.

How the Money is Spent.

In a special article Mr. Robert Hudson, chairman of the Joint Finance Committee, gives an account of stewardship. After touching upon the sources from which the money has come, he summarises the directions in which the major part of it has been spent. In stores alone there has been an expenditure of £218,000, in addition to clothing and comforts received by the Committee as gifts, and valued for the purposes of the accounts at £220,000. On the purchase, equipment, and running of the motor-ambulance fleet £450,000 has already been paid out, and in addition the Committee has further liabilities on this account of £131,000. In salaries and wages £265,000 has been paid out. King George Hospital has cost £39,000 in equipment, and in addition £500 a week is paid towards the salaries of the civilian staff of the hospital. These are a few of the main items of expenditure, but a better idea of the vastness of the Committee's undertakings can be obtained from the fact that of late the daily average expenditure has been £400.

